# First record of the genus *Lethades* Davis, 1897 from the Oriental region, with description of a new species (Hymenoptera, Ichneumonidae, Ctenopelmatinae)

**DOI:** 10.3897/zookeys.644.10491

**Published:** 2017-01-10

**Authors:** Alexey Reshchikov, Zai-fu Xu, Hong Pang

**Affiliations:** 1 College of Ecology and Evolution, Sun Yat-sen University, 135 Xingangxi St. Guangzhou, Guangdong 510275, China; 2 College of Agriculture, South China Agricultural University, Guangzhou, Guangdong 510640, China

**Keywords:** China, key, new species, Pionini

## Abstract

A new species of the genus *Lethades* Davis, 1897 (Ctenopelmatinae: Pionini), *Lethades orientalis* Reshchikov & Xu, **sp. n.**, collected in Heishiding Nature Reserve in Guangdong Province, China, is described. This is new record of the genus from China and for the Oriental region also. The species can be distinguished from all other species of the genus by its black metasoma, the presence of an areolet on the fore wing, distinctly pectinate claws with teeth of the hind claw as high as the claw itself, and a complete longitudinal propodeal carina. A key to the world species of the genus is provided.

## Introduction

The genus *Lethades* Davis, 1897, is in the tribe Pionini and the subfamily Ctenopelmatinae (Hymenoptera, Ichneumonidae). It formerly comprised 16 described species, two of them reported from the Nearctic Region and the rest from the Palaearctic Region (Yu et al. 2012).

Species of *Lethades* have been reared from Nematinae (Hymenoptera, Tenthredinidae) of the genera *Amauronematus*, *Nematus*, *Pachynematus*, and *Pristiphora* (Hinz 1961, 1976, 1996a, 1996b; Zinnert 1969; Pschorn-Walcher and Zinnert 1971). Only one species, *Lethades schaffneri* (Hinz) is known to attack *Rhadinocera eanodicornis* Konow, 1886 of the subfamily Blennocampinae (Hinz 1996a).

The European species of *Lethades* Davis were reviewed by Hinz (1996a), who provided a key to the Palaearctic fauna. Afterwards, Kasparyan and Khalaim (2007) developed a key to the species of the Russian Far East based on Hinz’s key. Two species, *Lethades alpinus* (Zetterstedt) and *Lethades flavifrons* (Zetterstedt) were synonymized with *Lethades curvispina* (Thomson) by Hinz (1996b) and *Lethades poloniae* Hinz, 1996 was synonymized with *Lethades punctatissimus* (Strobl) by Horstmann (2001). Cameron and Wharton (2011) transferred *Hodostates schaffneri* (Hinz) to *Lethades* based on ovipositor characters. In the Nearctic Region one species, *Lethades kukakensis* (Ashmead), is known from Alaska, and the other species, *Lethades texanus* (Ashmead) from Texas (Yu et al. 2012). One species, *Lethades buriator* Aubert, 1987 was described from The Republic of Buryatia (Eastern Russia) and five European species were recorded from the Eastern Palaearctic (Yu et al. 2012). Prior to this paper no species of either genus had been recorded from China or the Oriental Region. Here a new species is described from China, representing the first record of the genus from the Oriental region.

## Materials and methods

Specimens were collected using sweep nets in the forests of Heishiding Provincial Nature Reserve, located in Fengkai County, Zhaoqing City, West Guangdong Province, bordering Guangxi, China (23°27’N, 111°53’E, 150–927 m) (Zhang 1997). The reserve consists of subtropical evergreen and broad-leaved forests. The region has a subtropical moist monsoon climate with mean annual temperature 19.6°C and mean monthly temperatures range from 10.6°C in January to 28.4°C in July (Wang and Liu 1987). Annual precipitation is approximately 1743.8 mm, with rainfall occurring mainly between April and September (79% of annual total), there is a pronounced dry season lasting from October to March (Wang and Liu 1987). Species belonging to the Fagaceae and Lauraceae families, which are broadly distributed in subtropical evergreen broadleaved forests, are the dominant tree species (Chan et al. 2004).

The holotype is deposited in the Hymenopteran Collection of South China Agricultural University, Guangzhou (SCAU). Images were taken using AxioCamHRc digital camera attached to Zeiss Discovery V20 microscope and stacked using Helicon Focus®. All images were further processed using various minor adjustment levels in Adobe Photoshop®. Stacked images are available in colour and high resolution at http://www.morphbank.net. Morphological terminology mostly follows Gauld (1991). Wing vein nomenclature follows Ross (1936) and wing vein terminology follows Mason (1986, 1990).

## Taxonomy

### Lethades

Taxon classificationAnimaliaHymenopteraIchneumonidae

Davis, 1897

Lethades Davis, 1897: 204. Type species: Adelognathus texanus Ashmead, 1890. Monobasic.

#### Diagnosis.

*Lethades* can be distinguished from all other genera in the Pionini by the combination of the following characters: first flagellomere longer than second; second trochanter of hind leg rounded without a transverse ridge; glymma present; profile of the propodeum nearly rounded with short posterior field; dorsomedian and dorsolateral carinae of the T1 converging at base; ovipositor without subapical notch; cerci parallel-sided and protruding (Townes 1970, Cameron and Wharton 2011). The notaulus varies from absent to deep, but very short, in nearly all described species of *Lethades*. Only *Lethades schaffneri* Hinz has an elongate notaulus. The latter species was transferred to *Lethades* based on other characters, especially ovipositor morphology (Cameron and Wharton 2011).

#### Key to world species of the genus *Lethades*

**Table:** 

1	Notauli distinctly impressed extending at least over the anterior 0.5 of the mesoscutum. Claws not pectinate	***Lethades schaffneri* Hinz**
–	Notauli absent or weakly impressed, not extending the anterior 0.5 of the mesoscutum. Claws pectinate	**2**
2	Fore wing areolet absent	**3**
–	Fore wing areolet present	**5**
3	Body finely striated and weakly punctate. Fore femur and tibia uniformly red	***Lethades amauroneinati* (Hinz)**
–	Body distinctly and densely punctate, mesopleuron weakly striated. Fore femur and tibia not uniformly red	**4**
4	Epicnemial carina reaching anterior edge of mesopleuron. Metasomal tergites without yellow bands on posterior margins. Fore femur and tibia red, dark apically	***Lethades schmiedeknechti* Hinz**
–	Epicnemial carina not reaching anterior edge of mesopleuron. Metasomal tergites with yellow bands on posterior margins. Fore femur black, yellowish apically; fore tibia yellow	***Lethades texanus* (Ashmead)**
5	Metasomal tergites black, or with narrow posterior margins light colored (Fig. 1	**5**
–	Middle metasomal tergites red, sometimes with dark maculae	**11**
6	Claw distinctly pectinate, teeth of hind claw more than 0.5 times as high as claw (Fig. 7). Longitudinal propodeal carina absent or complete	**7**
–	Hind claw with teeth less than 0.5 times as high as claw. Longitudinal propodeal carina present	**8**
7	Longitudinal propodeal carina mostly absent, only the area apicalis defined	***Lethades punctatissimus* (Strobl)**
–	Longitudinal propodeal carina complete (Fig. 4)	***Lethades orientalis* Reshchikov & Xu, sp. n.**
8	Head with parallel sides or expanded behind eyes dorsally. T1 with distinct dorsal longitudinal carinae reaching almost to posterior margin	***Lethades erichsonii* Hinz, 1996**
–	Head narrowed behind eyes dorsally. T1 with weak dorsal longitudinal carinae reaching only 0.7 of length	**9**
9	Mesopleuron polished ventrally, finely and densely punctate. Propodeum with costula defined. T2 and T3 finely sculptured, polished. Metasomal tergites with narrow yellow posterior margins. Clypeus in female entirely or apically pale. Scape yellow ventrally	***Lethades cingulator* Hinz**
–	Mesopleuron matt ventrally, shagreened or granulated	**10**
10	T2 and T3 with broad reddish-yellow bands on posterior margins. Pronotum, mesonotum, and mesopleuron in male with large yellow maculae. Female with clypeus and scape entirely black	***Lethades laricis* Hinz**
–	T2 and T3 black (T3 slightly reddish-brown basally). Male with pronotum, mesonotum, and mesopleuron black. Clypeus in female with yellow maculae on sides	***Lethades buriator* Aubert**
11	Ovipositor sheath 2 times as long as first tarsomere of hind leg	***Lethades lapponicus* (Holmgren)**
–	Ovipositor sheath equal to or shorter than first tarsomere of hind leg	**12**
12	Temples and lower part of mesopleuron coriaceous and granulated with fine, dense punctures. T1 very densely punctate and striated, with elongate dorsal carinae reaching to its middle	***Lethades facialis* (Brischke)**
–	Temples and lower part of mesopleuron striated, punctures not defined. T1 finely shagreened, with short dorsal carinae not reaching middle	**13**
13	Ovipositor sheath curved upwards, as long as first tarsomere of hind leg, curved upwards. Antenna with 24–26 flagellomeres; the basal flagellomeres stout, and apical flagellomeres transverse. T1 black; T2–T4 red	***Lethades lapponator* Hinz**
–	Ovipositor sheath straight, shorter, 0.6–0.8 times as long as first tarsomere of hind leg. Antenna with 23–31 flagellomeres; the basal flagellomeres elongate, the apical flagellomeres cubic. T1 black with posterior margin red	**14**
14	Antenna with 28–31 flagellomeres. Female with third flagellomere 2.3–2.8 times as long as broad; male with third flagellomere 2.2–2.4 times as long as broad. Mesopleuron finely striated, finely and sparsely punctate. Body black. Palpi, mandibles, posterior edge of pronotum, and tegulae yellow. Legs (except coxae and hind tarsi), posterior margins of T1, and T2–T3 red. Male with clypeus, face, scape and pedicel ventrally, subtegular carina, fore and middle coxae and trochanters yellow	***Lethades imperfecti* Hinz**
–	Antenna with 23–28 flagellomeres. Female with third flagellomere 1.9–2.4 times as long as broad; male with third falgellomere 1.9–2.2 times as long as broad. Mesopleuron distinctly coriaceous	**15**
15	Mesonotum and T1–T3 finely striated	***Lethades scabriculus* (Thomson)**
–	Mesonotum and T1–T3 not striated	**16**
16	Mesonotum distinctly matt, finely and densely punctate	***Lethades kukakensis* (Ashmead)**
–	Mesonotum distinctly polished, sparsely and indistinctly punctate	***Lethades curvispina* (Thomson)**

### Lethades orientalis

Taxon classificationAnimaliaHymenopteraIchneumonidae

Reshchikov & Xu sp. n.

http://zoobank.org/D2D7BFCF-4430-4060-A53B-C52ECC0C0058

Figures 1–7

#### Type material.

*Holotype*, female, CHINA: Guangdong, Fengkai, Heishiding Provincial Nature Reserve (23°27'N, 111°53'E), 150–927 m., sweep net, 1–2.X.2003, leg. Zaifu Xu (SCAU).

#### Diagnosis.

**Figures 1–7.**
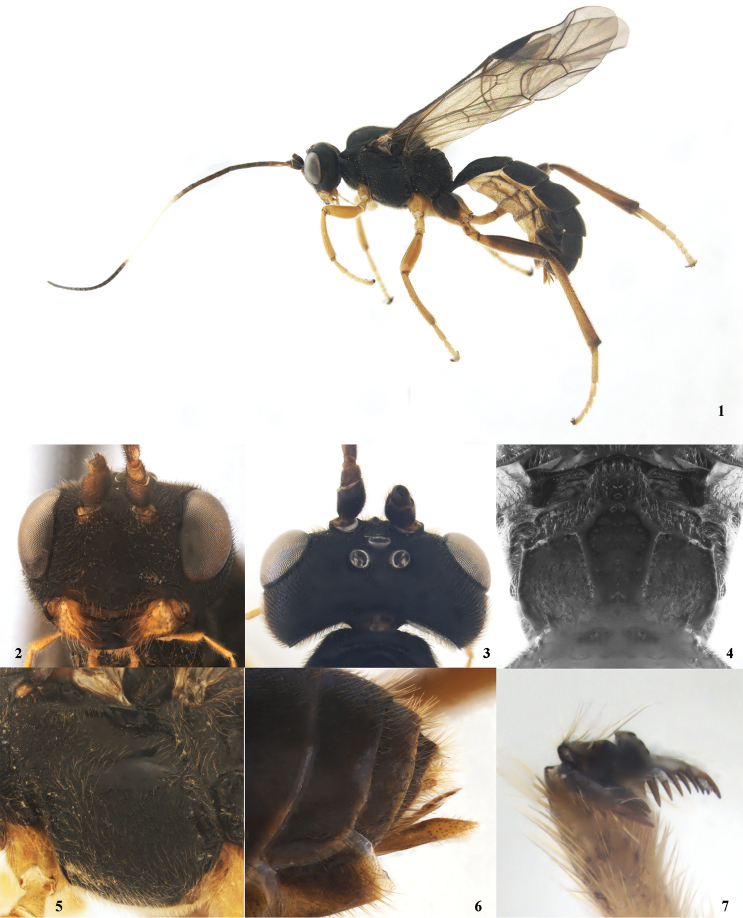
*Lethades orientalis* Reshchikov & Xu, sp. n., female, holotype. **1** Habitus, lateral view **2** head, frontal view **3** head, dorsal view **4** propodeum, dorsal view **5** mesopleuron **6** apex of metasoma, lateral view **7** claw.

This species can be distinguished from all other species of *Lethades* by a combination of the following characters: metasoma black (Fig. 1); fore wing with areolet; claw distinctly pectinate, teeth of hind claw as high as claw (Fig. 7); longitudinal propodeal carina complete (Fig. 4).

#### Description.

**Female.** Body length 10.5 mm.

*Head*. Face and clypeus shagreened and covered with long reddish setae (Fig. 2). Face approximately 1.4 times as wide as high, with distinct, sparse and shallow punctures; median portion weakly and longitudinally convex (Fig. 2). Clypeus convex, gradually raised towards apical margin, with very sparse, fine and distinct punctures. Upper tooth of mandible obviously shorter than lower tooth. Malar space approximately 0.6 times as long as basal width of mandible. Gena, vertex and frons shagreened. Head with parallel sides behind eyes (Fig. 3). Gena in lateral view approximately as long as the width of eye. Postocellar line nearly 0.5 times as long as ocular-ocellar line. Antenna with 40 flagellomeres. Occipital carina complete.

*Mesosoma*. Pronotum distinctly punctate. Mesoscutum with dense and distinct punctures. Notauli present. Scuto-scutellar groove with weak longitudinal wrinkles. Scutellum convex, with distinct punctures, its basal 0.3 with lateral carina. Mesopleuron (Fig. 5) and metapleuron coriaceous, mat, densely and distinctly punctate. Epicnemial carina distinct, its upper end reaching middle of mesopleuron. Wings slightly brownish, hyaline (Fig. 1). Fore wing with vein 1cu-a interstitial. Hind wing vein 1-cu approximately 1.9 times as long as cu-a. Hind femur 4.4 times as long as broad at its middle. Ratio of length of hind tarsomeres 37 : 14 : 11 : 6 : 16. Claw distinctly pectinate, teeth of hind claw as high as claw (Fig. 7). Propodeum rather short (Fig. 4). Its carinae strongly raised (Fig. 4). Area superomedia and area apicalis fused to form a pentagonal area, costula connecting in front of its middle (Fig. 4). Propodeal spiracle rounded.

*Metasoma*. Metasomal tergites shagreened, matt, finely punctate. T1 twice as long as apical broad. Dorsal carinae strongly raised, almost reaching posterior margin of T1. T2 transverse. Ovipositor sheath approximately 0.8 times as long as apical depth of metasoma, subapical portion distinctly truncated (Fig. 6). Ovipositor moderately stout, without distinct subapical notch (Fig. 6).

*Color*. Body mostly black (Figs 1–7). Mandibles, palpi, pedicel and basal part of first flagellomere ventrally, ovipositor sheath reddish. Flagellomeres 10–20 and apical tarsomeres white. Metasomal sternites and legs excluding hind coxa, femur, and distal and proximal parts of tibia reddish-yellow.

**Male.** Unknown.

#### Etymology.

The name of the new species refers to the Oriental Region.

## Supplementary Material

XML Treatment for Lethades

XML Treatment for Lethades orientalis

## References

[B1] Cameron MD Wharton RA (2011) Revision of *Hodostates* (Hymenoptera: Ichneumonidae: Ctenopelmatinae), with a discussion of tribal placement. Canadian Entomologist 143(2): 136–156. https://doi.org/10.4039/n10-054

[B2] Chan BPL Lau MWN Sai-Chit N Fellowes JR (2004) Report of a Rapid Biodiversity Assessment at Heishiding Nature Reserve, West Guangdong, China, July 2002. South China Forest Biodiversity Survey Report Series (Online Simplified Version): No. 39. KFBG, Hong Kong SAR, 19 pp.

[B3] Davis (1897) A review of the Ichneumonid subfamily Tryphoninae. Transactions of the American Entomological Society 24: 193−348.

[B4] Gauld ID (1991) The Ichneumonidae of Costa Rica, 1. Memoirs of the American Entomological Institute 47: 1−589.

[B5] Hinz R (1961) Über Blattwespenparasiten (Hym. und Dipt.). Mitteilungen der Schweizerischen Entomologischen Gesellschaft 34: 1–29.

[B6] Hinz R (1976) Zur Systematik und Ökologie der Ichneumoniden V. Deutsche Entomologische Zeitschrift 23: 99–105. https://doi.org/10.1002/mmnd.19760230111

[B7] Hinz R (1996a) Zur Systematikeiniger Ctenopelmatinae (Hymenoptera, Ichneumonidae). Nachrichtenblatt der Bayerischen Entomologen 45(3/4): 75–78.

[B8] Hinz R (1996b) Übersichtüber die europäischen Arten von *Lethades* Davis (Insecta Hymenoptera, Ichneumonidae, Ctenopelmatinae). Spixiana 19(3): 271–279.

[B9] Horstmann K (2001) Revision en von Schlupfwespen-Arten V (Hymenoptera: Ichneumonidae). Mitteilungen Münchener Entomologischen Gesellschaft 91: 77–86.

[B10] Kasparyan DR (1981) [A guide to the insects of the European part of the USSR. Hymenoptera, Ichneumonidae. 11 Ctenopelmatinae. 12 Phrudinae. 13 Tersilochinae. 14 Cremastinae. 15 Campopleginae. 16 Ophioninae.] Opredeliteli Faune SSSR 3(3): 316–431. [In Russian]

[B11] Kasparyan DR Khalaim AI (2007) Pimplinae, Tryphoninae, Eucerotinae, Xoridinae, Agriotypinae, Lycorininae, Neorhacodinae, Ctenopelmatinae, Phrudinae, Ophioninae, Acaenitinae, Collyriinae, Mesochorinae. In: Lelej AS (Ed.) Key to the Insects of Russia Far East (Vol. IV) – Neuropteroidea, Mecoptera, Hymenoptera (Pt 5). Dalnauka, Vladivostok, 474–559. [In Russian]

[B12] Mason WRM (1986) Standard drawing conventions and definitions for venation and other features of wings of Hymenoptera. Proceedings of the Entomological Society of Washington 88: 1–7.

[B13] Mason WRM (1990) Cubitus posterior in Hymenoptera. Proceedings of the Entomological Society of Washington 92: 93–97.

[B14] Pschorn-Walcher H Zinnert KD (1971) Investigations on the ecology and natural control of the larch sawfly (*Pristiphora erichsonii*) (Hym.: Tenthredinidae) in central Europe. Part II. Natural enemies: their biology and ecology, and their role as mortality factors in *P. erichsonii*. Commonwealth Institute of Biological Control Technical Bulletin 14: 1–50.

[B15] Ross HH (1936) The ancestry and wing venation of the Hymenoptera. Annals of the Entomological Society of America 29: 99–111. https://doi.org/10.1093/aesa/29.1.99

[B16] Townes HK (1970) The genera of Ichneumonidae, Part 3. Memoirs of the American Entomological Institute 13(1969): 1–307.

[B17] Wang B Liu X (1987) The Characteristics of the Vegetation in Hei Shi Ding Natural Reserve. Ecologic Science 1/2: 1–18. [In Chinese with English abstract]

[B18] Yu DS Achterberg C van Horstmann K (2012) World Ichneumonoidea 2011. Taxapad 2012. Vancouver.

[B19] Zhang J (1997) Nature Reserves of Guangdong Province. Guangdong Tourism Publishing House, Guangzhou, 384 pp [In Chinese]

[B20] Zinnert KD (1969) Vergleichende Untersuchungenzur Morphologie und Biologie der Larven parasiten (Hymenoptera Ichneumonidae und Braconidae) mitteleuropäischer Blattwespenaus der Subfamily Nematinae (Hymenoptera: Tenthredinidae). Teil I. ZeitschriftfürAngewandte Entomologie 64: 180–217. https://doi.org/10.1111/j.1439-0418.1969.tb03036.x

